# Ketamine Does Not Produce Relief of Neuropathic Pain in Mice Lacking the β-Common Receptor (CD131)

**DOI:** 10.1371/journal.pone.0071326

**Published:** 2013-08-01

**Authors:** Maarten Swartjes, Marieke Niesters, Lara Heij, Ann Dunne, Leon Aarts, Carla Cerami Hand, Hyung-Suk Kim, Michael Brines, Anthony Cerami, Albert Dahan

**Affiliations:** 1 Department of Anesthesiology, Leiden University Medical Center, Leiden, The Netherlands; 2 Araim Pharmaceuticals, Ossining, New York, United States of America; 3 University of North Carolina at Chapel Hill, Chapel Hill, North Carolina, United States of America; University of Arizona, United States of America

## Abstract

Neuropathic pain (NP) is a debilitating condition associated with traumatic, metabolic, autoimmune and neurological etiologies. Although the triggers for NP are diverse, there are common underlying pathways, including activation of immune cells in the spinal cord and up-regulation of the N-methyl-D-aspartate receptor (NMDAR). Ketamine, a well-known NDMAR antagonist, reduces neuropathic pain in a sustained manner. Recent study has shown that the novel 11-amino acid peptide erythropoietin derivative ARA290 produces a similar, long-lasting relief of NP. Here, we show that both drugs also have similar effects on the expression of mRNA of the NMDAR, as well as that of microglia, astrocytes and chemokine (C-C motif) ligand 2, all-important contributors to the development of NP. Although the effects of ketamine and ARA 290 on NP and its molecular mediators suggest a common mechanism of action, ARA 290 has no affinity for the NMDAR and acts specifically via the innate repair receptor (IRR) involved in tissue protection. We speculated therefore, that the IRR might be critically involved in the action of ketamine on neuropathic pain. To evaluate this, we studied the effects of ketamine and ARA 290 on acute pain, side effects, and allodynia following a spared nerve injury model in mice lacking the β-common receptor (βcR), a structural component of the IRR. Ketamine (50 mg/kg) and ARA 290 (30 µg/kg) produced divergent effects on acute pain: ketamine produced profound antinociception accompanied with psychomotor side effects, but ARA290 did not, in both normal and knock out mice. In contrast, while both drugs were antiallodynic in WT mice, they had no effect on NP in mice lacking the βcR. Together, these results show that an intact IRR is required for the effective treatment of NP with either ketamine or ARA 290, but is not involved in ketamine’s analgesic and side effects.

## Introduction

Neuropathic pain (NP), arising from lesions affecting the somatosensory system [1], is often not adequately treated by current pharmacotherapy, such as antidepressants, opioids, and topical agents (e.g., lidocaine or capsaicin). It is a common feature following trauma or infectious, autoimmune, metabolic, and neurological diseases [2], [3], and is often accompanied as well by hyperalgesia. Inflammation arising from injury plays an important role in the development and maintenance of NP and the peripheral and central sensitization phenomena that establish allodynia and hyperalgesia depend upon a variety of neuromodulatory processes [2]. These may include the activation and up-regulation of dorsal horn excitatory glutamatergic N-methyl-d-asparate receptors (NMDAR), as well as a vigorous inflammatory response within the spinal cord initiated and maintained by microglia and reactive astrocytes as well as the production of TNF-α, interleukins and CCL2 (reviewed in ref. [4]).

Recently, evaluation of preclinical models of NP have shown that erythropoietin (EPO) is locally produced following peripheral nerve injury and functions as an endogenous factor that limits damage and improves nerve function [5], [6]. The tissue protective effects are mediated by the EPO receptor-ß-common receptor complex [7], rather than the EPO receptor homodimer (EPOR_2_) involved in erythropoiesis. This isoform, termed the innate repair receptor (IRR), may additionally form functional complexes with other molecules to transduce specific cellular responses (e.g. vascular endothelial growth factor receptor-2 or endothelial nitric oxide synthase, reviewed in ref. [8]). Generally, inflammation and tissue injury induce both the expression of the IRR and the production of local EPO in a characteristic temporal and spatial pattern (reviewed in ref. [9]).

Treatment of NP in animal models with exogenous EPO results in relief of allodynia and hyperalgesia and attenuates a number of detrimental cellular responses, including neuronal apoptosis and pro-inflammatory cytokine production while enhancing beneficial cellular responses, including regeneration and anti-inflammatory cytokine production. Recently, we have shown that the novel EPO-derivative ARA 290, specifically interacting with the IRR and not with the EPOR_2_ thus rendering it without hematopoietic side effects, is able to persistently relieve NP [10].

Accordingly, recent clinical trials of ARA 290 in patients with neuropathy have shown benefit [11], [12]. Similar observations have been made following treatment of patients with NP with ketamine, which has shown to have potent and long-lasting analgesic effects on NP [13]–[15]. However, ketamine is associated with significant side effects that limit its use outside of closely monitored medical settings. Ketamine acts at multiple receptor systems in addition to antagonism of the NMDAR. Evidence also suggests it also possesses anti-inflammatory activities and inhibits the activation of microglia and astrocytes which play prominent roles in the development of NP [16], [17]. The similarity of action of ARA 290 and ketamine on NP raised the hypothesis that both compounds share a common mechanistic pathway, possibly involving the IRR. To evaluate this possibility, we compared the effects of ketamine and ARA 290 on the expression of the NMDAR subunits, glia cell markers, and the signaling molecule CCL2 in a NP pain model. Further, since receptor gene knock out studies constitute a powerful method to establish roles of specific receptor systems in complex biological responses [18], we also compared the differences in nociception and psychomotor effects, known to depend upon NMDAR, and NP behavior elicited by sciatic nerve injury in wild type mice and to ßcR^−/−^ mice that lack responses activated by the IRR.

## Materials and Methods

### Ethics

The study protocols were approved by the animal ethics committee of the Leiden University Medical Centre and the Animal Care and Use Review Office (ACURO) of the United States Army Medical Department Medical Research and Materiel Command. All experiments were performed according to the guidelines of the International Association for the Study of Pain [19].

### Animals

Six to eight week-old female C57Bl/6 mice were purchased from Charles River, Maastricht, The Netherlands. ß-common-receptor knockout mice (ßcR^−/−^) were obtained from The William Harvey Research Institute, London, UK. Confirmation of the genotype was done as described by Robb et al. using Southern blot analysis [20]. All animals were housed in groups of 4–5 per individually ventilated cage with water and food available at libitum and a 12 h light-dark cycle (lights on/off at 7AM/7PM).

#### Drugs

ARA 290 (Araim Pharmaceuticals, New York, USA) and ketamine (Eurovet, Bladel, The Netherlands) were dissolved to yield a 30 µg/kg and 50 mg/kg dose in a 200 µl injection volume and were administered intraperitoneally. All drugs were dissolved in PBS (vehicle).

### *In vitro* Screening Assay

ARA 290 (10 µg) was evaluated in the “High Throughput Profile” of CEREP, Inc. (Poitiers, France) and the N-methyl-D-aspartate binding assay as described at www.cerep.fr. No significant interaction of ARA 290 with any of the screens was observed (data not shown).

### QRT-PCR

To establish a profile of the transcriptional changes of the mRNA of specific cytokines and receptors induced by the SNI or and the effect of the investigated drugs on those cytokines, QRT-PCR was performed on tissue of the injured sciatic nerve and spinal cord. Naïve mice (n = 5) served as reference for basal mRNA expression levels. Mice that had received SNI, with or without treatment (n = 5/group), were sacrificed 7 days post lesion. The nucleotide sequences of the PCR primers and their fluorogenic probes for the target genes were designed by using the computer program primer express (PE Biosystems) and are included in Table 1. Each fluorescent probe has a reporter dye (FAM for the target RNA and TET for the 18S RNA control) covalently attached at its 5′ end and a quencher dye (TAMRA) attached at its 3′ end. Before use, the probes were purified in the PolyPak II cartridge (Glen Research, Sterling, VA) following the manufacturer’s instructions. RNA was isolated from the sciatic nerve and spinal cord from each of 5 mice in the experimental groups outlined above with the ABI Prism 6100 Automated Nucleic Acid Workstation according to the manufacturer’s protocol. Real-time RT-PCR amplifications were employed as described in ref. [21]. The numbers of copies of the PCR template in the starting sample were calculated by using the sequence detector software incorporated in the ABI Prism 7300 Sequence Detector System. Sense RNAs were synthesized from the standard plasmids by the manufacturer’s protocols, using a MAXIscript transcription kit (Ambion). The concentrations of purified sense RNAs were determined as micrograms per optical density unit, and serial dilutions of the sense RNA, using bacterial tRNA as a carrier, were used to generate standard curves. When quantification was relative to an endogenous control, standard curves were prepared for both the target and the endogenous control. We assumed that 18S RNA is present in all tested and control samples of tissue RNA at a constant proportion and normalize the amount of total RNA in our test samples by comparing their 18S RNA fluorescent signal after PCR with that from mouse embryonic stem cell RNA freed from DNA by DNase treatment. Relative mRNA levels are expressed as using 18S RNA as reference.

**Table 1:** QRT-PCR primers and probes used in this study.

Gene	Type	Sequence (5′–3′)
Grin1(NMDAR NR1)	Forward	GTC CAT CTA CTC TGA CAA GAG
	Reverse	AAA CCA GAC GCT GGA CTG GT
	Probe	f TCC ACC TGA GCT TCC TTC GCA CCGq
Grin2a(NMDAR NR2A)	Forward	ACC TCG CTC TGC TCC AGT TT
	Reverse	GTT GTG GCA GAT GCC CGT AA
	Probe	f CAG TGT CTC CAG CTC TTC CAT CTC ACq
Grin2b(NMDAR NR2B)	Forward	TGG TCT TCT CCA TCA GCA GA
	Reverse	GTT CAT CAC GGA TTG GCG CT
	Probe	f ATC TAC AGC TGT ATC CAC GGA GTA GCq
CCL2	Forward	CTG GAG CAT CCA CGT GTT G
	Reverse	TGG GAT CAT CTT GCT GGT GA
	Probe	f AGC CAG ATG CAG TTA ACG CCC CAC T q
AIF-1 (Iba1)	Forward	GCA ATT CCT CGA TGA TCC CA
	Reverse	ATG TAC TTC ACC TTG AAG GCT
	Probe	f CAG CAA TGA TGA GGA TCT GCC GTC CAq
GFAP	Forward	CTC AAG AGG AAC ATC GTG GT
	Reverse	TGC TCC TGC TTC GAG TCC TT
	Probe	f TGA CCT CAC CAT CCC GCA TCT CCAq
18S	Forward	AGA AAC GGC TAC CAC ATC CA
	Reverse	CTC GAA AGA GTC CTG TAT TGT
	Probe	tAGG CAG CAG GCG CGC AAA TTA Cq

Primers and probes used for the quantification of mRNA from NMDA receptor subtypes NR1, NR2A and NR2B (Grin); microglia marker Iba-1 (AIF-1), astrocyte (GFAP) and CCL2; f, Reporter dye1 (FAM:6-carboxyfluorescein); t, Reporter dye2 (TET:Tetrachloro-6-carboxyfluorescein); q, Quencher dye (TAMRA: 6-carboxytetramethyl1-rhodamine).

### Acute Antinociception

In uninjured animals (n = 5/treatment group), tail withdrawal latencies (TWL) were recorded to determine the antinociceceptive effect of the drugs. The water bath was heated to 47.5°C which resulted in a baseline response with a threshold of 9–11 seconds. The tail of the mouse was immersed in water and the latency to withdraw the tail was recorded. A cut-off value of 30 s was used to prevent tissue damage. Baselines were recorded prior to injection of the drugs and TWL were recorded 30 and 60 minutes after injection. TWL were obtained in duplicate with an interval of 30 seconds in between measurements and averaged.

### Side Effects

Side effects induced by drug treatment (n = 5/treatment group) were assessed in uninjured animals by using a method adapted from ref. [22]. Briefly, animals were observed for 60 min post injection at 5-min intervals. Stereotypic behavior was scores on a 7-point scale as: -3: anesthesia, -2: sedation, -1: drowsiness, 0: normal, 1: moderately increased (increased explorative behavior), 2: increased (increased urge to move around the cage), 3: greatly increased (inability to hold still with weaving, shaking or twitching of the head and body). Activity level was defined as follows: -3: anesthesia, -2: sedation, -1: drowsiness, 0: normal, 1: moderately impaired (disturbances in paw support), 2: impaired (unable to maintain paw support with the ability to regain an upright position after falling over), 3: greatly impaired (inability to regain an upright position after falling over).

### Spared Nerve Injury

Mice (n = 8/treatment group per genotype) were anesthetized with isoflurane (4% induction and 2% maintenance) and were operated to receive a spared nerve injury (SNI) as described previously [10]. In short, a lateral incision on the left thigh was made, exposing the muscle. The left sciatic nerve was then exposed by blunt preparation and the tibial and common peroneal nerves were ligated with 6–0 silk sutures, transected and displaced to prohibit any regeneration. Consecutively, muscle integrity was restored and the wound was closed with 5–0 sutures. In case of sham SNI, animals were anesthetized and the sciatic nerve was exposed as described above. After exposure no SNI was induced and the wound was closed. Animals were administered a single s.c. injection of 0.1 mg/kg buprenorphine for the relief of acute post operative pain and were allowed to recover from surgery in a clean cage with body temperature maintained at 38°C and were observed for 1 h before being transferred back to the cage with fresh sawdust. The animals were followed up for 7 days or 42 days.

#### Tactile allodynia

Assessment of tactile allodynia was performed using Semmes-Weinstein monofilaments. Animals were placed in transparent Perspex cages on a grid and allowed to habituate to the experimental environment for 5–10 min. After habituation, filaments were applied to the ipsi-lateral hind paw by applying 10 stimulations over 10 s. Failure to respond led to progression to the next filament exerting a greater force. Withdrawal of the stimulated paw led to the recording of the force of the corresponding filament. All measurements were done in duplicate with a 30 second interval between measurements and averaged.

### Statistical Analysis

All behavioral data was analyzed for a treatment effect by two-way repeated measures analysis of variance (ANOVA) followed by a post hoc Student-Newman-Keuls test for multiple comparisons. QRT-PCR data was analyzed by one-way ANOVA followed by a post hoc Student-Newman-Keuls test for multiple comparisons when distributed normally. In the absence of a normal distribution, as defined by the Shapiro-Wilk criterion, or unequal variance, data was analyzed by a Kruskal-Wallis one-way ANOVA on ranks followed by a Student-Newman-Keuls test for multiple comparisons. p-values <0.05 were considered significant. Analysis was done with SigmaPlot version 12 (SyStat Software, Inc. Chicago, USA).

## Results

### Ketamine and ARA 290 Attenuate Neuropathy-related mRNA Changes of the Spinal Cord in a Similar Manner

**Figure 1:**
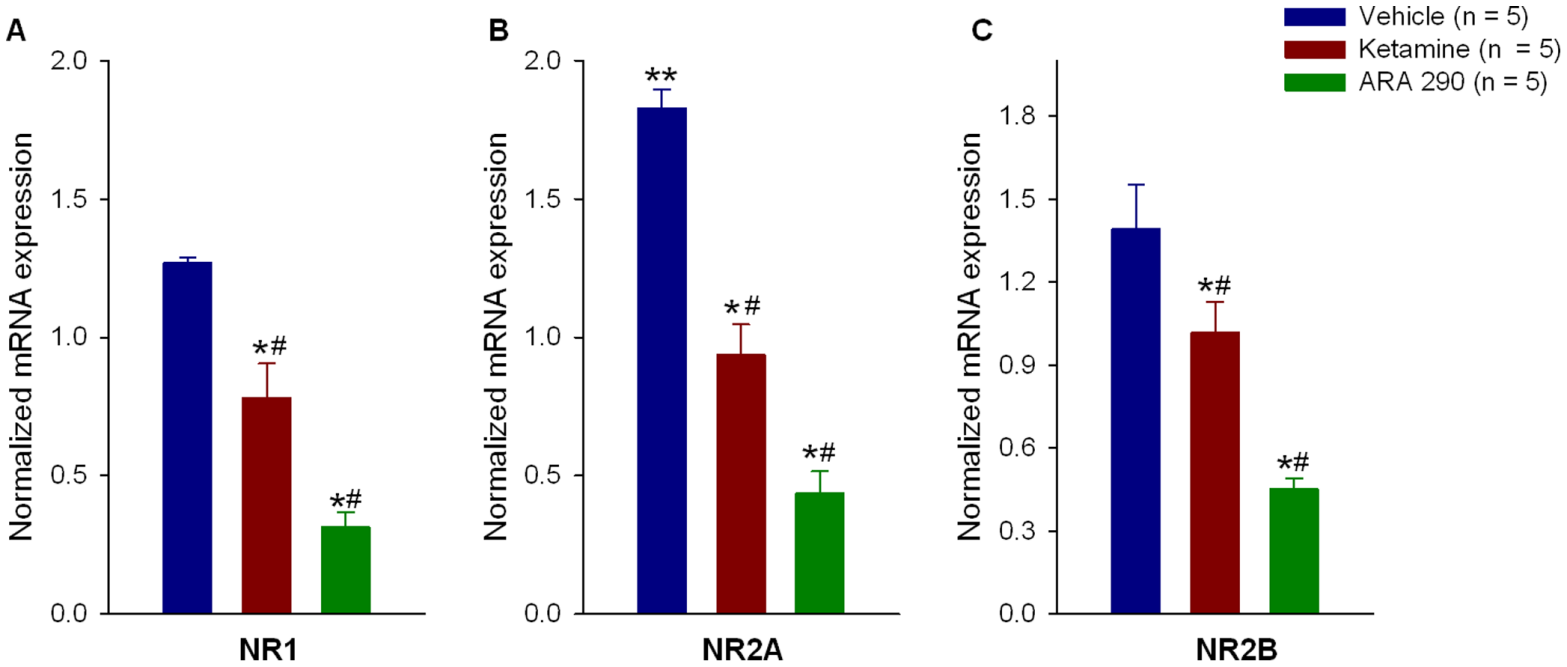
Ketamine and ARA 290 reduce mRNA for NDMA receptor subunits in established neuropathy. Real time PCR data show that NMDA receptor subunits 1 (panel **A**), 2A (panel **B**), and 2B (panel **C**) are all modestly elevated one week following sciatic nerve injury. Administration of ketamine significantly reduces mRNA to baseline levels. In contrast, ARA 290 reduced mRNA for these receptor subunits to substantially below baseline (naïve). *p<0.05 *versus* vehicle, #p<0.05 between ketamine and ARA 290 treatments, **p<0.05 *versus* naïve.

To evaluate changes in gene expression for potentially relevant receptors and inflammatory molecules in the development of allodynia, animals were sacrificed on day 7 following sciatic nerve injury and real time PCR performed on extracts of the spinal cord. SNI with vehicle treatment moderately changed NMDAR subunit mRNA expression of NR1 (1.27±0.02 fold, p = 0.183), NR2A (1.83±0.07 fold, p<0.001) and NR2B (1.39±0.16 fold, p = 0.101) when compared to naïve (uninjured and untreated) animals at 7 days post injury (Figs. 1A–C). Treatment with either drug significantly decreased expression of NR1, NR2A and NR2B mRNA when compared to injured, vehicle treated animals. For NR1 mRNA, treatment resulted in changes in expression of 0.78±0.13 fold (p = 0.042 *versus* vehicle) and 0.31±0.05 fold (p = 0.002 *versus* vehicle) for ketamine and ARA 290, respectively, with ARA 290 inducing the greater changes in mRNA levels (p = 0.022 between treatments). For NR2A mRNA, changes in expression of 0.94±0.11 fold (p<0.001 *versus* vehicle) and 0.44±0.08 fold (p<0.001 *versus* vehicle) were observed for ketamine and ARA 290 respectively, with ARA 290 inducing the greater changes in mRNA levels (p = 0.007 between treatments). The mRNA levels of NR2B after treatment were 1.02±0.11 fold (p = 0.048 *versus* vehicle) and 0.45±0.04 fold (p = 0.002 *versus* vehicle) for ketamine and ARA 290 respectively, with ARA 290 inducing the greater changes in mRNA levels (p = 0.019 between treatments).

**Figure 2:**
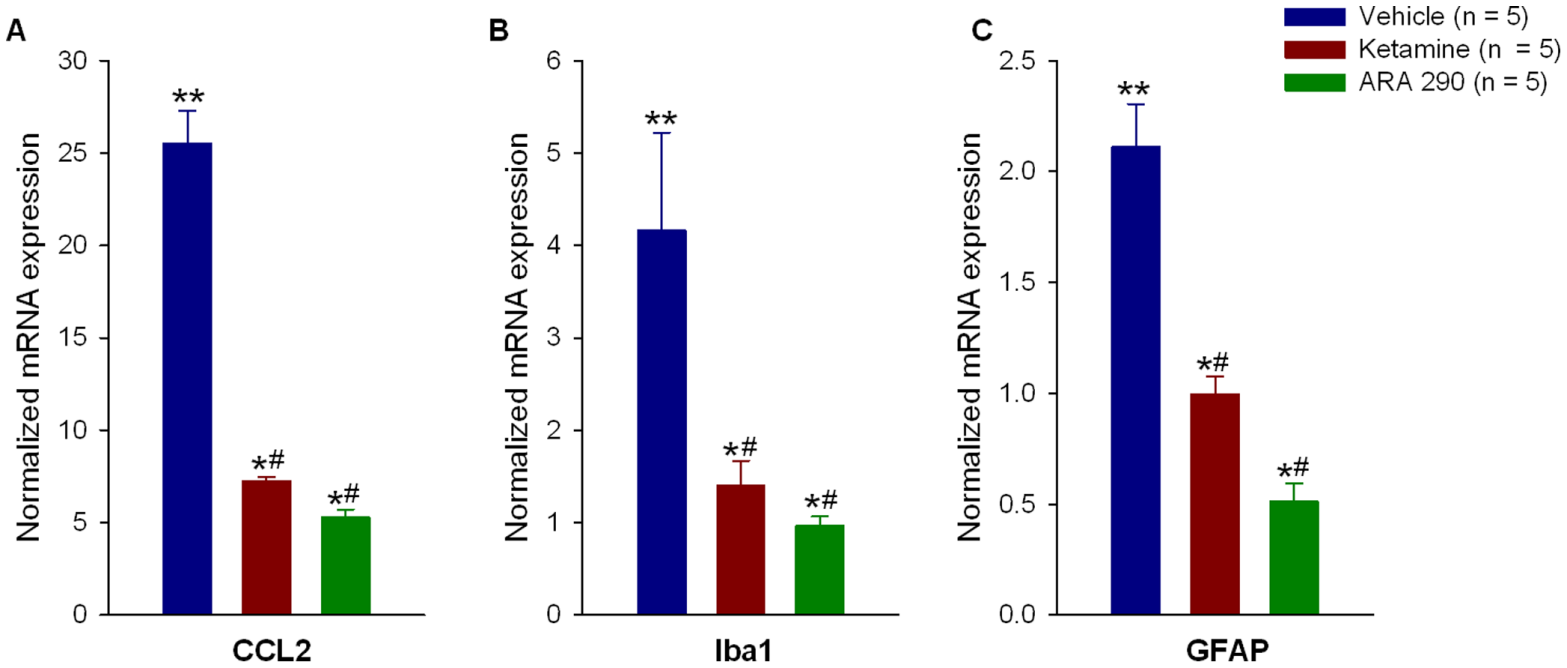
Ketamine and ARA 290 reduce inflammatory mediators in the spinal cord following sciatic nerve injury. One week post surgery, animals showed a marked elevation of CCL2 (panel **A**), Iba1 (panel **B**), and GFAP (panel **C**) compared to naïve controls. Both ketamine and ARA 290 significantly reduced the mRNA levels of these genes to a similar extent. *p<0.05 *versus* vehicle, #p<0.05 between ketamine and ARA 290 treatments, **p<0.05 *versus* naïve.

The microglial response to SNI followed by vehicle treatment (mediated by chemokine (C-C motif) ligand 2 (CCL2), also known as macrophage chemotactic protein 1 (MCP-1)) showed a significant 25.53±1.8 fold increase in CCL2 mRNA relative to naïve animals (p<0.05), which was attenuated by ketamine (7.26±0.29 fold, p<0.05) and ARA 290 (5.27±0.44 fold, p<0.05), with ARA 290 inducing a greater change in mRNA levels (p<0.05 between treatments, Fig. 2A). SNI followed by vehicle treatment significantly increased the microglia activation marker ionized calcium binding adaptor molecule 1 (Iba-1) mRNA by 4.16±1.06 fold (p = 0.01 *versus* naïve). Both treatments significantly decreased Iba-1 mRNA to 1.41±0.27 fold (p = 0.008 *versus* vehicle) and 0.96±0.10 fold (p = 0.015 versus vehicle, Fig. 2B) for ketamine and ARA 290 respectively. Ketamine and ARA 290 were equally effective when comparing between treatments (p = 0.839). The increase in the astrocyte marker glial fibrillary acidic protein (GFAP) mRNA following SNI and vehicle treatment (2.11±0.20 fold, p<0.001) was significantly reduced by ketamine (0.99±0.08 fold, p = 0.001) and ARA 290 (0.51±0.08, p<0.001, Fig. 2C). Treatment with ARA 290 induced greater changes in mRNA when comparing between treatments (p = 0.039).

### Ketamine and ARA 290 have Divergent Effects on Acute Nociceptive Pain and Behavior

**Figure 3:**
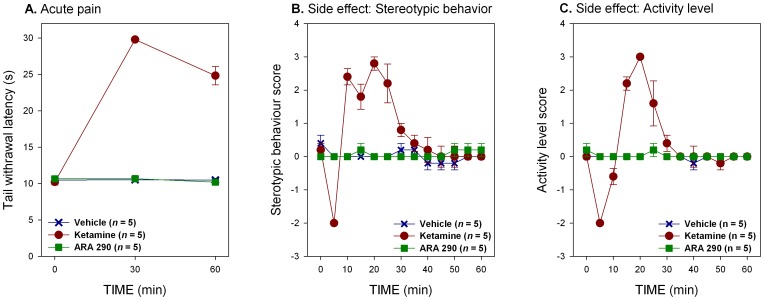
Ketamine and ARA 290 differ in effects on acute nociceptive pain and side effects. **A.** Ketamine administration increases the latency of tail withdrawal to a thermal stimulus (treatment effect, p<0.001), whereas ARA 290 does not. **B.** Ketamine treatment had significant biphasic effects on stereotypic behavior: after a period of transient sedation, the animals showed signs of psychomimetic disturbances that lasted for about 20 minutes (treatment effect, p<0.001). ARA 290 did not display these side effects. **C.** Treatment with ketamine was associated with a biphasic activation of generalized activity (treatment effect, p<0.001) causing an increase in restlessness and explorative behavior after a period of transient sedation.

Normal mice exhibited a brisk withdrawal response within 9–11 s after tail immersion in heated water of 47.5°C (Fig. 3A). Vehicle treated animals did not show alterations in TWL during the follow up period after injection. Ketamine administration (50 mg/kg intraperitoneally) rapidly induced an acute antinociceptive effect as demonstrated by a marked increase in the withdrawal latency (TWLs of 29.8±0.16 s and 24.85±1.26 s for 30 and 60 min post injection respectively, treatment effect p<0.001 versus vehicle, Fig. 3A). In contrast, animals administered ARA 290 (30 µg/kg i.p.) demonstrated no change in latency during the 60 min observation period following administration (treatment effect p = 0.977 *versus* vehicle, Fig. 3A). Additionally, ketamine administration was associated with side effects characterized by the induction of stereotypical behavior and changes in locomotor activity. Following ketamine, administration mice displayed a period of transient sedation which dissipated within 10 min, followed by a longer excitatory state characterized by stereotypical behavior and increased locomotor activity that lasted for 20–25 min before subsiding (treatment effects for stereotypical behavior and activity level, p<0.001 and p<0.001 *versus* vehicle respectively, Figs. 3B and C). In contradistinction, no behavioral changes were observed following ARA 290 (treatment effects for stereotypical behavior and activity level, p = 0.549 and p = 0.346 *versus* vehicle respectively, Figs. 3B and C).

### Ketamine and ARA 290 do not Effect Allodynia in Mice Lacking the ß-common Receptor

**Figure 4:**
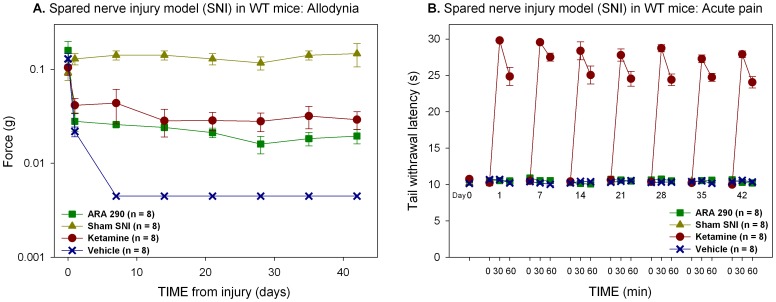
Ketamine and ARA 290 have similar effects on allodynia. **A.** Treatment with both ketamine and ARA 290 prevented the full development of allodynia (treatment effect, p = 0.049 and p = 0.03, respectively). **B.** The effects of ketamine on acute nociceptive pain remained unchanged over time (treatment effect, p<0.001).

**Figure 5:**
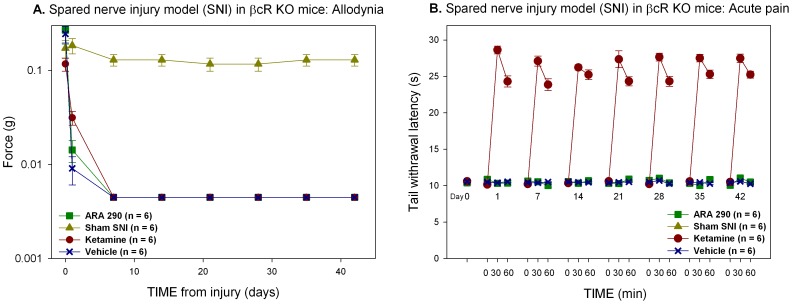
Relief of allodynia depends upon the ß-common receptor (ßcR). **A.** Both ketamine and ARA 290 did not prevent the development of allodynia in ßcR^−/−^ mice. **B.** However, the effect of ketamine on nociceptive pain is unchanged in ßcR^−/−^ animals (treatment effect, p<0.001).

Following sciatic nerve surgical transection in which the sural branch is preserved (spared nerve injury; SNI), tactile allodynia developed in both wild type and ßcR^−/−^ mice within 24 h as demonstrated by significant decreases in the force required to induce a withdrawal response following plantar stimulation (Figs. 4A and 5A). To evaluate potential effects of ARA 290 and ketamine on the development of allodynia, animals were administered drug or vehicle every other day beginning 24 hours following surgery, and weekly starting in the second week after surgery. Following injury, vehicle treated animals uniformly displayed allodynia, reaching a nadir in the force required to induce a withdrawal within 7 days at an applicable force of 0.004±0.0 g. Allodynia was sustained for the duration of the follow up. In C57Bl/6 (wild type; WT) mice, treatment with either ARA 290 (30 µg/kg i.p.) or ketamine (50 mg/kg i.p.) significantly attenuated the development of tactile allodynia during the first week, which was maintained for the duration of the follow up period (treatment effect versus vehicle, p = 0.049 and p = 0.03 for ketamine and ARA 290 respectively, Fig. 4A). Contrastingly, both ARA 290 and ketamine were ineffective in sciatic nerve transected ßcR^−/−^ mice, (treatment effect *versus* vehicle, p = 0.308 and p = 0.730 for ketamine and ARA 290, respectively, Fig. 5A), although ketamine retained its acute antinociceptive effect (Figs. 4B and 5B) and behavioral effects (data not shown) similar to those observed in WT animals.

## Discussion

The results of these experiments show that (1) nerve injury coincides with changes in expression levels of NMDAR subunit mRNA and spinal cord inflammatory mediators (CCL-2, Iba-1 and GFAP) after 7 days of NP, which are blunted by both ketamine and ARA 290; (2) ketamine induces relief of acute nociceptive pain and behavioral side effects, whereas ARA 290 lacks these acute effects; (3) ketamine and ARA 290 induce relief of allodynia (*i.e.,* analgesia) in a similar manner in wild type mice, but have no effect in mice lacking the ßc chain. Contrastingly, ßcR^−/−^ status had no effect on the acute antinociceptive or psychomotor effects of ketamine.

As would be predicted if ketamine and ARA 290 shared a common mechanism of action on NP, examination of gene expression in the spinal cord of injured animals shows comparable effects of these two agents on NMDA receptor expression and spinal cord inflammatory marker levels. The development of NP is associated with up-regulation of NMDAR on neurons that are believed to interact with microglia and astrocytes within the dorsal root ganglia and the dorsal horn of the spinal cord ipsilateral to the injured nerve [23]. Surprisingly, seven days following sciatic nerve injury mRNA of the NMDAR subunits NR1, NR2A and NR2B are not markedly elevated above the levels observed in uninjured animals, despite the fact that the maximum measurable amount of allodynia was reached. Similar observations are described in 2 other models of NP, where a significant NMDA receptor upregulation did not occur until after 14 days of NP following spinal cord injury for NR1, NR2A and NR2B [24], or where a reduction of the NMDA receptor at peak levels of allodynia was observed [25]. In our study, ketamine treatment significantly reduces the mRNA of all the NMDA receptor subunits to expression levels comparable to those of naïve animals, in contrast to the slightly elevated levels of untreated controls. In comparison, ARA 290 has a significantly larger suppressive effect and markedly reduced gene expression of the NMDARs below expression levels observed in naïve animals. Dose-response analyses were not performed in this study; therefore it is uncertain whether this difference between ARA 290 and ketamine can be explained by potency or biological factors. In spite of unequal NMDAR mRNA suppression by these agents, the effect of both on allodynia is identical. Taken together the slightly increased expression of the NMDA receptor subunits with respect to the amount of allodynia and the identical effect of both drugs on allodynia further support the possibility that the contribution of NMDAR to allodynia may not be the principal determinant in the development in NP, which has been suggested by the results of an earlier study [26].

Both ketamine and ARA 290 induce an approximately equivalent suppression of microglia, astrocyte and CCL2 mRNA measured within the spinal cord. The activation of spinal cord cells such as microglia and astrocytes is correlated to NP and reducing the numbers or activation states of these cells has shown to be of importance for the reduction of allodynia [27]. Iba1 as a marker for activated microglia and the suppression of its mRNA to baseline levels is consistent with significant attenuation of inflammation within the spinal cord and the subsequent reduction in allodynia. In addition to the reduction of microglia, the observed reduction of astrocytes as identified by GFAP mRNA may also have contributed to the anti-allodynic effect of both ketamine and ARA 290. Notably, CCL2 is a product of neurons within the effected region of the spinal cord and signals for the accumulation of microglia in NP [28]. Antagonism of the C-C chemokine receptor type 2 (CCR2), the target receptor of CCL2, has shown to decrease allodynia [28], [29]. CCL2 signaling and glia cell activation in conjunction with NMDAR up-regulation have been considered to be hallmarks of central sensitization [4], the observed effects of both ARA 290 and ketamine on these effectors is consistent with a reduction of central sensitization and with the observed results of these compounds on NP behavior.

Ketamine and ARA 290 have shown different effects on acute nociception that can be explained by the different receptor targets of the drugs. Specifically, the NMDAR, which mediates glutamate-dependent pain signaling arising from depolarization of the afferent nerve fibers, is antagonized by by ketamine, but not by ARA 290. Therefore, blocking NMDAR activity by using 50 mg/kg ketamine results in a profound relief of acute nociceptive pain, whereas treatment with ARA 290 does not. Moreover, treatment with 50 mg/kg ketamine coincides with psychomimetic and locomotor side effects where mice suffer a transient period of sedation, as classified by a reduced activity level and subsequent explorative (stereotypical) behavior, followed by a hyperactive state. Treatment with ARA 290 did not induce these side effects. These results indicate that ketamine, but not ARA 290, interacts with the NMDAR. In contrast to the data on nociceptive pain, both drugs attenuate the development of allodynia following nerve injury in a similar manner. When administered to WT animals 24-h after lesion, tactile allodynia is persistently decreased in both ketamine and ARA 290 treated animals during the entire follow up period. In addition, the analgesic action of ketamine does not change during SNI status. Conversely, ßcR^−/−^ mice with an SNI that were treated with ketamine or ARA 290 did not benefit from treatment and no attenuation of allodynia was observed. However, ketamine was still able to induce acute antinociception in mice lacking the ßcR. The ßcR requires assembly with other receptor subunits to become a functional signaling unit. As ARA 290 only interacts with the ßcR-EPOR heteromer (IRR), it is a distinct possibility that the action of ketamine in this model is also mediated through the IRR. Ketamine possesses strong anti-inflammatory properties and is able to reduce serum TNF-α after sepsis in a murine laparoscopic model, an effect not readily expected from ketamine’s action on the NMDA receptor [30]. Furthermore, it has been noted in the SNI model that NMDA receptor blockade by MK 801 did not affect either mechanical or cold allodynia [26].

ARA 290 has a very short plasma half-life (∼2 minutes [31]) while ketamine has a tissue half-life of 10–12 minutes [32], [33]. In spite of this, both agents have sustained effects on pain behavior in the spared nerve model. *In vivo* nerve recording has demonstrated that the antiallodynic effects of ketamine are maintained far longer than its NMDAR antagonism [33]. ARA 290 and ketamine administered every 48 hours for the first 5 doses and weekly thereafter prevent the development of allodynia and sustain this (anti-allodynic) state in spite of chronic nerve injury. This observation is consistent with a modulating effect in which brief exposure to these pharmacologic agents activates a molecular switch, to produce long term effects through changes in gene expression. Previously a beneficial effect on pain behavior has been noted for treatment with amitriptyline extending to beyond the elimination half-life and treatment period in the very same model we have employed [34]. This effect is similar to the long-term modulation in the nervous system activity (“plasticity”), which underlies learning, memory and the development of NP. The observation that ketamine may affect NP behavior by use of a signaling pathway that includes a receptor that is a component of the innate immune response and repair system predicts that it may also affect other functions served by this receptor, such as beneficial effects on inflammation at multiple levels, including the recruitment of immune-competent cells and secretion of pro-inflammatory cytokines and chemokines (reviewed in ref. [35]).

**Figure 6:**
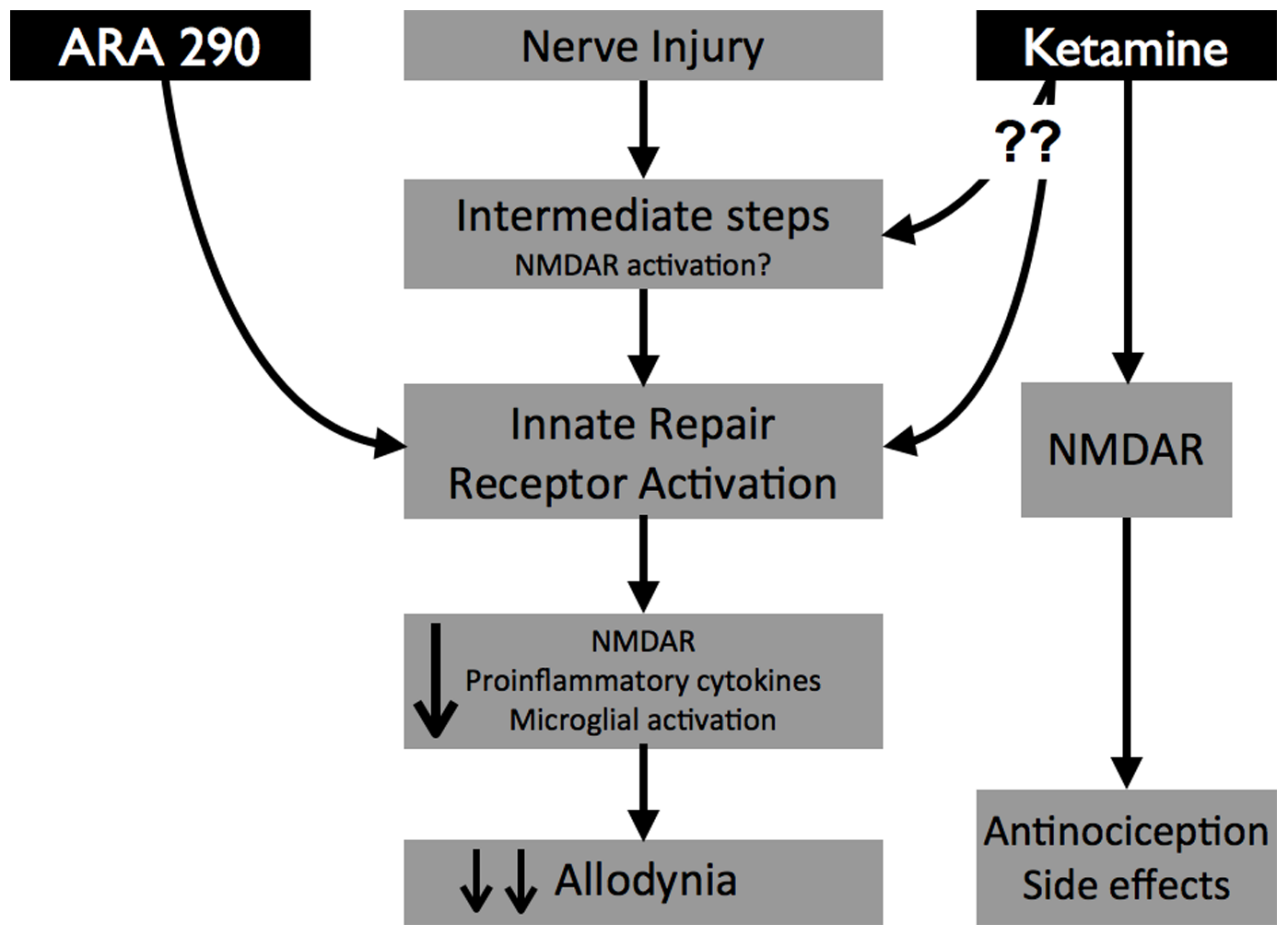
Neuropathic pain involves a pathway that utilizes the Innate Immune Receptor. Nerve injury results in microglial recruitment, increased expression of NMDAR, and proinflammatory cytokine production, ultimately resulting in allodynia. Activation of the innate immune receptor (IRR), e.g., by ARA 290, antagonizes this pathway. Ketamine also requires the IRR to reduce allodynia. This may be via a direct interaction with the IRR or alternatively, via modulation of intermediate processes that are upstream of the IRR. Additionally, ketamine interacts with NMDARs that mediate antinociception and psychomotor effects. ARA 290 does not interact with the NMDAR and therefore lacks these additional effects.

In conclusion, these findings confirm the existence of a pathway in the evolution of NP that involves the IRR (Fig. 6). Ketamine has a distinct effect on acute nociceptive pain, but a different activity in common with ARA 290 on NP via a pathway that requires the ßcR. Whether the observed effects depend upon a direction interaction of ketamine with the IRR with require further studies, e.g., receptor binding or NMDAR knockdown experiments. The similar effects of ketamine and ARA 290 on NP in the spared nerve model established through activity of the IRR, could also be true for other models of NP, as well as for other treatments of NP. Although the effects of ketamine on acute pain appear to be pharmacologically driven by interaction by the NMDA receptor, the long-term effects may be described as manipulating a molecular switch, altering downstream gene expression and subsequent detrimental effects. Finally, although ketamine has potent, long lasting anti-neuropathic effects, interaction with NMDARs leads to very significant adverse effects including abuse potential. Utilization of IRR specific ligands, e.g., ARA 290, avoids these undesirable effects and may point a way to improved therapy of neuropathic disease.
